# Reemergence of St. Louis Encephalitis Virus in the Americas

**DOI:** 10.3201/eid2412.180372

**Published:** 2018-12

**Authors:** Adrián Diaz, Lark L. Coffey, Nathan Burkett-Cadena, Jonathan F. Day

**Affiliations:** Universidad Nacional de Córdoba, Córdoba, Argentina (A. Diaz);; University of California, Davis, California, USA (L.L. Coffey);; University of Florida, Vero Beach, Florida, USA (N. Burkett-Cadena, J.F. Day)

**Keywords:** St. Louis encephalitis virus, emerging infectious disease, phylogeography, arbovirus, viruses, United States, Americas, South America

## Abstract

We summarize and analyze historical and current data regarding the reemergence of St. Louis encephalitis virus (SLEV; genus *Flavivirus*) in the Americas. Historically, SLEV caused encephalitis outbreaks in the United States; however, it was not considered a public health concern in the rest of the Americas. After the introduction of West Nile virus in 1999, activity of SLEV decreased considerably in the United States. During 2014–2015, SLEV caused a human outbreak in Arizona and caused isolated human cases in California in 2016 and 2017. Phylogenetic analyses indicate that the emerging SLEV in the western United States is related to the epidemic strains isolated during a human encephalitis outbreak in Córdoba, Argentina, in 2005. Ecoepidemiologic studies suggest that the emergence of SLEV in Argentina was caused by the introduction of a more pathogenic strain and increasing populations of the eared dove (amplifying host).

## Abstract 2

In the western United States, this virus may have been mediated via migrating infected birds from southern South America, where it reemerged most recently in 2002.

The disease known as St. Louis encephalitis (SLE) is caused by St. Louis encephalitis virus (SLEV), identified as the causative agent of a mosquitoborne viral epidemic in St. Louis, Missouri, USA, during the summer of 1933 (*1*). SLEV is transmitted by numerous mosquito species in the genus *Culex* and is amplified by passerine and columbiform avian species (*1*). Phylogenetic analysis based on the full-length E gene sequences grouped SLEV strains into 8 genotypes (*2*). Genotypes I and II are prevalent in the United States and genotype V is widely distributed in South America. Other genotypes have limited distribution: genotype III is in southern South America, IV is limited to Colombia and Panama, VI is in Panama, VII is in Argentina, and VIII has been detected only in the Amazon region of Brazil (*2*).

A retrospective analysis revealed that 38 human cases and 14 deaths were caused by SLEV in Paris, Illinois, USA during the summer of 1932 (*3*). A 1933 SLE epidemic resulted in 1,095 clinical human cases and 201 deaths (*3*). Because subclinical cases are not identified or reported, retrospective serosurveys were conducted to determine the ratio of subclinical to clinical infections, which was determined to be 300:1 (*3*). Using this ratio, the actual number of SLE cases during the 1933 SLE epidemic was ≈328,500, affecting nearly 40% of the city’s 821,960 inhabitants, based on US census data for 1930 (*4*).

Since SLEV was first identified, 4 human SLE transmission scenarios have been reported (*5*). First, during most years, no human SLE infections are reported. However, SLEV transmission to sentinel animals and virus isolation from mosquito pools is documented in the absence of human cases. Second, small numbers of spatially and temporally isolated human SLE cases occur. For example, in 1993, 8 human SLE cases were reported in Lee and Collier counties, Florida, USA; 5 of the cases reported onset during October (*6*). Third, sporadic transmission occurs as widely dispersed (temporarily and spatially) individual human cases. For example, in 1997, 9 human SLE cases were reported from 6 Florida counties, ranging from Brevard County on the central Atlantic Coast south to Lee County on the southern Gulf Coast. Onset for these cases ranged from July through late October (*6*). Finally, epidemic transmission occurs as focused (in space and time) clusters of human clinical cases: for example, an extensive 1975 epidemic that occurred along the Mississippi and Ohio River basins from Ontario, Canada; Cleveland, Ohio; and Chicago, Illinois, in the north to Birmingham, Alabama, and Mississippi in the south. Well-documented SLE outbreaks include the 1933 St. Louis epidemic and the 1959, 1961, 1962, 1977, and 1990 epidemics in south Florida. Other epidemics of note occurred in St. Louis (1937); Hidalgo County, Texas (1954); High Plains, Texas; Louisville, Kentucky; and Cameron County, Texas (1956); and Houston, Texas (1964). There was also a 1990 SLE epidemic in Florida (*5*), and, most recently, a 2015 epidemic in the Phoenix area of Arizona (Figure 1).

**Figure 1:**
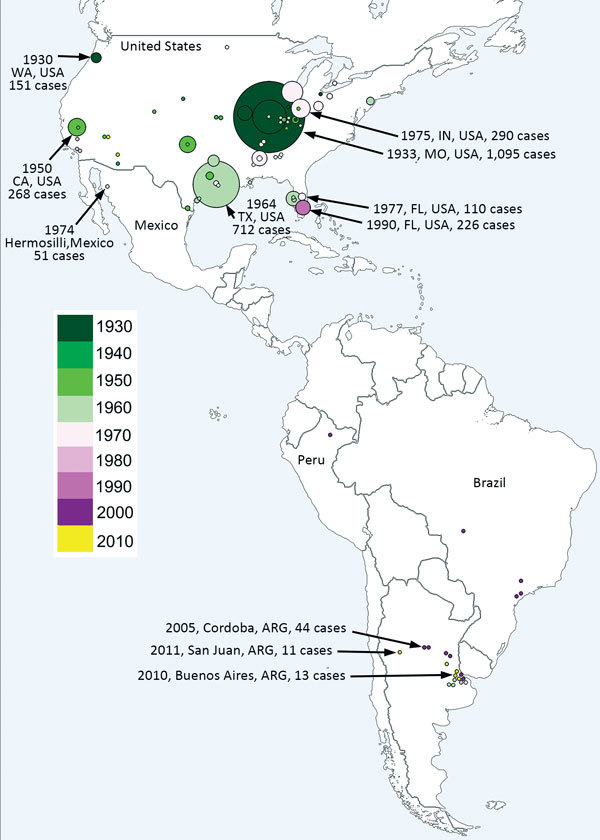
Geographic distribution of historical St. Louis encephalitis human cases reported in the Americas through November 2017. Dot size represents the number of human cases reported in each episode. Colors represent year of detection.

Epidemics of SLE are promoted by environmental factors including summer temperature, rainfall, snowmelt, and surface water conditions (*7*,*8*). One of the most notable environmental drivers for SLEV activity is the cycling of rainfall and drought. The wet–dry cycle can affect the epidemiology of SLEV by forcing gravid floodwater *Culex* vectors to delay oviposition long enough to complete viral development (extrinsic incubation) in a single gonotrophic cycle, thus making them capable of viral transmission during their second blood meal (*8*). Drought has also been linked to urban SLE and West Nile virus (WNV) epidemics involving vectors in the *Cx*. *pipiens* complex (*9*).

## Transmission Cycles

The 4 primary vectors of SLEV in the United States are *Cx. pipiens pipiens* Linnaeus, *Cx. pipiens quinquefasciatus* Say, *Cx. tarsalis* Coquillett, and *Cx. nigripalpus* Theobald mosquitoes (*1*). Vector species distribution determines the geographic distribution of SLEV, and affects whether epidemics are urban or rural. Urban SLE epidemics usually involve *Cx. pipiens pipiens* and *Cx. pipiens quinquefasciatus* (*Cx. pipiens* complex) mosquitoes, species that oviposit in the permanent aquatic habitats provided by storm drains, sewage treatment facilities, and wastewater retention ponds. Rural SLE epidemics usually involve floodwater species, such as *Cx*. *tarsalis* and *Cx*. *nigripalpus *mosquitoes (*1*). Urban and rural epidemic transmission patterns are best demonstrated by the history of SLEV in Florida, where urban human SLEV cases were detected in Miami in 1952 and 1954, followed by St. Petersburg in 1959, Tampa in 1961, and Sarasota in 1962. In 1977, SLEV epidemic transmission in Florida shifted from urban to rural areas (*7*). The 1977 and 1990 Florida SLE epidemics both started in Indian River County and then spread throughout the Florida peninsula (*7*). The temporal shift from urban to rural epidemic transmission in Florida was facilitated by changes in *Cx. nigripalpus* oviposition preference and behavior. The *Cx. nigripalpus *mosquito is a widespread subtropical species and a highly opportunistic blood feeder that oviposits in freshly flooded temporary aquatic habitats. In urban habitats, *Cx. nigripalpus *mosquitoes oviposit in wastewater retention ponds and open wastewater outflow ditches (*10*). The shift to rural transmission in 1977 and 1990 was facilitated by an increase in citrus farming, as citrus groves were designed to be maintained by flood irrigation (*10*). In the 1970s through the 1990s, rural citrus grove drainage furrows became the preferred oviposition site for *Cx. nigripalpus *mosquitoes (*11*). The proclivity of *Cx. nigripalpus *mosquitoes to blood-feed on birds as well as mammals and the tendency of females to delay oviposition until the proper aquatic oviposition habitats are created by heavy summer rainfalls make it an excellent vector of SLEV (*11*).

The introduction of WNV into the United States in 1999 promoted debate about how the presence of WNV would affect the continued transmission of SLEV, given the serologic cross-reactivity of the 2 viruses in avian hosts. Laboratory studies demonstrated that house finches (*Haemorhous mexicanus*) experimentally infected with WNV developed neutralizing immunity that prevented infection after rechallenge with WNV or SLEV (*12*). In contrast, house finches first exposed to SLEV showed lower subsequent viremias after rechallenge with WNV. This suggests that WNV could competitively exclude SLEV from amplification in shared avian hosts like house finches (*13*). Indeed, transmission of SLEV in Florida decreased notably following the introduction and establishment of WNV (*13*). For competitive exclusion to suppress SLEV transmission, considerable host overlap between SLEV and WNV must occur. In Florida, avian species in the families Cardinalidae (northern cardinal, *Cardinalis cardinalis*), Columbidae (mourning dove, *Zenaida macroura*), Corvidae (blue jay, *Cyanocitta cristata*) and Icteridae (common grackle, *Quiscalus quiscula*) are frequently exposed to SLEV (*14*). For WNV, house sparrows (*Passer domesticus*) and blue jays are highly infectious to mosquitoes; in contrast, mourning doves were found to be 1 of the least competent WNV hosts of 25 bird species examined experimentally (*15*). Even if mourning doves are poor WNV hosts, being exposed to WNV may provide cross neutralization to SLEV and preempt amplification of SLEV, provided that doves are exposed to WNV early in the transmission season. In California, sparrows, finches, and corvids (house finch, house sparrow, purple finch [*Haemorhous purpureus*]); song sparrow (*Melospiza melodia*); western scrub jay (*Aphelocoma californica*); and white-crowned sparrow (*Zonotrichia leucophrys*) are considered major amplifying hosts of WNV and SLEV (*1*,*16*). As in Florida, host overlap of WNV and SLEV likely contributed to the initial disappearance of SLEV from the western United States (*17*). Of these avian hosts, only house sparrows, house finches, American crows (*Corvus brachyrhynchos*), and western scrub jays were found to be amplifying hosts capable of developing viremias above the threshold for infection of *Cx. tarsalis*, the principal vector of WNV and SLEV throughout much of California (*16*). Further work is needed to characterize the specific avian hosts fed on by *Cx. pipiens quinquefasciatus* and *Cx. tarsalis *mosquitoes in epidemic and nonepidemic settings to determine whether additional host species may be involved in amplification of SLEV and to what degree host overlap contributes to the competitive exclusion or reemergence of SLEV in areas of the United States outside California.

Since the introduction of WNV into the United States, human SLE cases continue to occur throughout the country. During 2004–2013, 92 clinical SLE cases were reported (*18*). Most cases were located in the Gulf Coast states of Louisiana (10 cases), Mississippi (*13*), and Texas (*16*). During 2014–2016, a total of 32 human SLE cases were reported (*19*). Most of these cases were reported in the Phoenix area, where a SLE epidemic resulted in 23 confirmed human cases, including 1 fatality (*19*). SLEV has remained endemic throughout much of the United States despite the introduction and establishment of WNV.

## Emergence and Reemergence in South America

Historically, SLEV has not been considered a major public health threat in the Americas, other than in the United States. Human SLE cases have been reported sporadically throughout Latin America, but no human epidemics were reported until 2005. In Argentina, Charosky et al. (*20*) reported a neurologic human SLEV case in 1968. Two years later, Mettler et al. (*21*) isolated SLEV from a febrile human suspected to have Argentinean hemorrhagic fever. Human encephalitis cases were reported sporadically until 2005, when an unprecedented outbreak of SLE was reported in Córdoba City, Argentina (*22*). During this outbreak, signs and symptoms associated with a neurologic infection, including headache, sensory depression, temporal–spatial disorientation, tremors, and changes in consciousness, were reported. A correlation between age and sign or symptom severity was detected (Spearman coefficient = 0.74) (*22*). The frequency of encephalomyelitis varied from 80% of the cases in patients <20 years of age to 95% in those >60 years of age (*22*). A total of 47 probable and confirmed human cases were reported. Of these, 45 patients were hospitalized; 9 died, 1 25 years of age and 8 >50 years of age (*22*).

The SLEV strains CbaAr-4006 and CbaAr-4006 were isolated from *Cx. pipiens quinquefasciatus *mosquitoes collected during the outbreak in the backyard of the index case-patient in Córdoba City (*23*). Molecular classification and phylogenetic analyses indicated that the strains isolated during the outbreak were closely related to a genotype III SLEV strain (79V-2533) that had been isolated in Santa Fe Province, Argentina (*23*). A 3-year retrospective phylogenetic analysis of SLEV genomes from mosquitoes collected in Córdoba City, designed to find an enzootic progenitor of the outbreak strains, indicated no circulation of genotype III before the 2005 outbreak (*24*). The extensive sampling effort detected low levels of SLEV transmission of genotypes other than genotype III. The CbaAr-4005 strain was more virulent and produced higher viremias in avian and murine models. In house sparrows, CbaAr-4005 produced viremias that were 2.2 logs higher and lasted for 2 days longer than its closest relative, the 1978 79V-2533 strain. Because mosquitoes show a dose response to infection, house sparrows inoculated with SLEV strain CbaAr-4005 will theoretically produce viremia sufficient to infect 10 times more mosquitoes than those inoculated with a nonepidemic strain (*25*).

**Figure 2:**
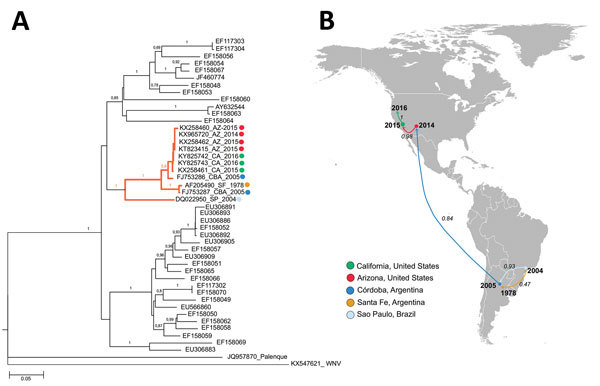
Phylogeny and spread of St. Louis encephalitis virus (SLEV) in the Americas. A) Multiple sequence alignment of 44 complete envelope protein SLEV sequences obtained from GenBank. The orange highlighted cluster contained the emerging SLEV strains isolated in Argentina, Brazil, and western United States. Alignment was performed by ClustalX, followed by tree generation using a neighbor-joining algorithm using MEGA 6 software (https://www.megasoftware.net*)*. Sequences are labeled by their GenBank accession number. Sequences belonging to lineage III also contain place and year of isolation data. Bootstrap support values are given for each node. Scale bar represents nucleotide substitutions per site. B) Geographic spread of SLEV. A discrete Bayesian phylogeographic reconstruction for SLEV lineage III was made using 11 envelope protein sequences (highlighted cluster in Figure 2, panel A). We applied a constant-size coalescent tree before the phylogeny and a TNF93 nt substitution model. The Monte Carlo Markov chain model was obtained after 30 million iterations and subsampling every 20.000 iterations. Analyses were made using Beast version 1.8.3 software (http://tree.bio.ed.ac.uk/software/beast/). Numbers over the arrows indicate the probability for the postulated viral dispersion in that pathway. Color of arrows indicates the location origin for the strain introduced.

In adult Swiss mice, the CbaAr-4005 strain of SLEV resulted in 100% (10/10) illness and death compared with sympatric nonepidemic SLEV strains isolated previously in Córdoba and Santa Fe Province, Argentina (*26*). Inoculation of only 1 plaque-forming unit in 10-day-old mice or 2 plaque-forming units in 21-day-old mice caused a 50% death rate with the strain CbaAr-4005 (lethal dose 50 [LD_50_] in 10-day-old mice = 0.02), proving it to be the most lethal strain compared in the study (78V-6507_LD50_ = 1.75; CorAn-9275_LD50_ = 3.90). That evidence supports the hypothesis that a more virulent SLEV strain was introduced into Córdoba City in early 2005 from Santa Fe (Argentina) or São Paulo (Brazil) (Figure 2). Serologic studies of wild birds sampled before and during the 2005 outbreak indicated that 99% (434/437) of the avian population lacked a SLEV-neutralizing antibody and were therefore susceptible to infection with SLEV, potentially serving as amplification hosts (*27*). The absence of natural immunity in the wild avian population may have promoted the 2005 SLE outbreak in Córdoba City. A host competence study in avian species showed that the eared dove (*Zenaida auriculata*) and picui ground-dove (*Columbina picui*) produce high levels of viremia compared with other avian species tested (*28*). Moreover, that study confirmed the role as amplifying host of these 2 doves species during the 2005 SLEV outbreak in Córdoba. Additional studies indicate that the population of eared doves has been increasing in the central region of Argentina during the past 10 years because of agricultural geographic expansion (*29*).

After the 2005 outbreak, additional SLE outbreaks in Argentina occurred in Parana (2006), Buenos Aires (2010), and San Juan (2011) (Pan American Health Organization, http://www.paho.org/hq/dmdocuments/2010/alert_epi_2010_31_marz_encefalitis_san_luis.pdf). Unfortunately, no ecologic studies focused on these outbreaks. Phylogenetic analysis of SLEV genomes associated with human cases in Buenos Aires confirmed the presence of SLEV genotype III (*30*).

SLE was diagnosed in a febrile human in São Paulo State, Brazil, in 2004, by viral isolation and molecular detection (*31*). Molecular characterization classified the isolate as SLEV genotype III. During a dengue virus outbreak in São José do Rio Preto (São Paulo State, Brazil) in 2006, Mondini et al. (*32*) reported the first outbreak of SLE reported in Brazil. Human cases were diagnosed by molecular detection of SLEV RNA in serum or cerebrospinal fluid. All SLEV-infected patients (*6*) had an initial diagnosis of dengue fever or viral encephalitis; 3 cases were diagnosed as viral meningoencephalitis, and the other 3 patients had signs of hemorrhagic disease (*32*). This finding was the first reported link of SLE infection and human hemorrhagic disease (*32*). A blastn analysis (https://blast.ncbi.nlm.nih.gov/Blast.cgi) comparing genomes of different SLEV strains, which we carried out for this review, indicates that the SLEV strains from Brazil are closely related to SLEV genotype V. This finding indicates that genotype V SLEV strains are also pathogenic for humans, as shown in previous studies in murine models (*26*). Because genotype V strains are widespread in South America, human SLEV cases could be misdiagnosed as dengue virus infection. A detailed analysis of South America human SLE cases is shown in Figure 1.

## Reemergence in Western United States

SLEV was recognized in 1937 in California; cases were documented during 1940–1950 and in Kern County during a 1952 outbreak of Western equine encephalitis virus (*33*). SLEV caused periodic epidemics in humans and epizootics in equines during the 1950s–1990s. However, expansive epidemics similar to those observed in Missouri and Texas were not detected in California, although concurrent activity in mosquitoes and seroconversions in birds were repeatedly documented in the San Joaquin Valley, Los Angeles Basin, and Imperial Valley (*1**,**34**,**35**,**36*). An outbreak resulting in 26 human cases occurred in 1984 in Los Angeles, (*34*), leading to avian studies (*37*), vector competence experiments (*38*), and vectorial capacity studies (*39*). A SLEV serosurvey following the Los Angeles outbreak found that 1.6% of 1,803 serum samples tested from residents of Los Angeles were seropositive. A subsequent outbreak of 26 confirmed SLEV cases was centered in Kern County but disappeared the following summer (*35*,*36*). Envelope gene sequencing from mosquito isolates suggested that different SLEV strains were most likely responsible for these outbreaks (*40*). Sequencing of longitudinal SLEV isolates from California showed that isolates were genetically similar from 1952 through the 1970s, after which genetic changes were observed (*41*).

Even in the absence of epidemics since 1989, SLEV activity was documented continuously in California from the 1990s through 2003 by human cases, detections in mosquitoes, or seroconversions in sentinel chickens (*42*). In 2003, WNV activity was first detected in the state (*17*). During 2003–2015, no SLEV activity was detected in California, despite ongoing SLEV surveillance in *Culex* mosquitoes and sentinel chicken seroconversions (*17*). The absence of SLEV activity during that period was likely not the result of a lack of surveillance because the invasion of WNV led to a ≈6-fold statewide increase in mosquito pool testing, from ≈5,000 pools in 2003 to 30,000–35,000 pools annually since 2007. Instead, the absence of SLEV activity suggested extirpation from California.

Beginning in July 2015, SLEV activity was detected by the presence of viral RNA in mosquito pools and sentinel chicken seroconversions in the Coachella Valley of Riverside County, California (*43*). In 2016, SLEV spread northward to 6 additional counties in California (*44*); in 2017, a total of 15 California counties reported activity (*45*). A clinical study used unbiased clinical testing by metagenomic next-generation sequencing to diagnose a fatal case of meningoencephalitis from SLEV in a patient from Kern County, California, in September 2016 (*46*).

In Arizona, SLEV detection was historically less frequent than in California; low enzootic activity was reported most years during 1972–2006 and only a single human case during 2010–2014 (*47*). In Maricopa County (which includes Phoenix), a human SLEV outbreak during July–October 2015 resulted in 23 confirmed cases (*48*). Three patients in that outbreak were organ transplant recipients who experienced fever, rigors, diarrhea, headache, and confusion; all developed meningoencephalitis, and 1 patient died (*48*). SLEV infection in the 2 surviving transfusion recipients was confirmed by plaque reduction neutralization tests (*48*). Retrospective testing of archived mosquito pools from Phoenix collected in 2014 revealed a single SLEV isolate, indicating that SLEV was present in Arizona the summer before the 2015 outbreak (*48*).

To define the genetic relatedness of reemerging SLEV from Arizona and California to SLEV from elsewhere to infer a possible origin and pattern of spread, we performed phylogenetic analyses of genomes from mosquitoes in 2014–2016 and the fatal case in 2016 in Kern County. The 2014 and 2015 California and Arizona SLEV isolates share >99% nucleotide identity with each other and also with their closest published relative isolated from *Cx. pipiens quinquefasciatus *mosquitoes collected in the 2005 epidemic in Córdoba City (*49*). The SLEV genome sequence from the fatal case in Kern County from September 2016 was >99% identical with 2014–2015 SLEV isolates from mosquitoes in California and Arizona, suggesting that the patient was infected by the reemergent genotype circulating in the southwestern United States (*46*). The 2014 and 2015 SLEV isolates are genetically distinct from the 2003 Imperial Valley, California, strain that was isolated before the 11-year absence of SLEV activity in the state (*49*). These results suggest there was likely a single introduction of SLEV into the United States from South America, and possibly Argentina, no later than November 2014, the earliest dated sample from which SLEV was isolated in Arizona and that the virus spread in the summer of 2015 from Arizona to California (*49*).

## Conclusion and Future Perspectives

Arthropodborne virus infections are emerging and reemerging infectious diseases worldwide. Mosquitoborne viruses, including dengue (emerged in 1990), WNV (emerged in 1999), chikungunya (2013), and Zika viruses (2015), have emerged as public health threats in the Western Hemisphere. SLEV is maintained in a bird–mosquito transmission network involving multiple species in a broad range of ecosystems encompassing a wide geographic distribution that ranges from southern Canada to southern Argentina. Biotic factors, including vector and host abundance and population age structure, as well as abiotic factors, including rainfall and drought dynamics and elevated summer temperatures, combine to produce conditions favorable for transmission of SLEV. A better understanding of how SLEV circulates between enzootic transmission cycles in nature and epidemic transmission in human populations is needed to more accurately predict where and when human SLEV epidemics will emerge.

The reemergence of SLEV in central Argentina is associated with several factors, including the recent introduction of a more virulent strain of SLEV into a naive bird community and increased populations of eared doves, a highly susceptible amplification species in Argentina. Argentina has experienced intense land use changes primarily because of the expansion of agricultural and urbanized habitats. More research is needed to define the effects of environmental change on avian reservoir and vector populations to clarify the dynamics of SLEV transmission, introduction, reintroduction, and reemergence in susceptible habitats throughout the Western Hemisphere.

The reemergence of SLEV in California and Arizona resulted from introduction of a South American strain of SLEV. The genetic diversity of SLEV in the Americas is spatially influenced, with wide genetic variation across the space, but some SLEV strains from North and South America show high genetic similarity, indicating long-range dispersal. Similar to WNV, long-range SLEV dispersal is likely mediated by migrating SLEV-infected birds. A better understanding of SLEV in wild birds and avian host migration patterns is necessary to predict the spread of SLEV.

## References

[1] Reisen WK. Epidemiology of St. Louis encephalitis virus. Adv Virus Res. 2003;61:139–83. 10.1016/S0065-3527(03)61004-3 14714432

[2] Rodrigues SG, Nunes MR, Casseb SM, Prazeres AS, Rodrigues DS, Silva MO, et al. Molecular epidemiology of Saint Louis encephalitis virus in the Brazilian Amazon: genetic divergence and dispersal. J Gen Virol. 2010;91:2420–7. 10.1099/vir.0.019117-0 20592112

[3] Chamberlain RW. History of St. Louis encephalitis. In: Monath TP, editor. St. Louis encephalitis. Washington (DC): American Public Health Association; 1980. p. 680.

[4] Monath TP, Tsai TF. Flaviviruses. In: Richman DD, Whitley RJ, Hayden FG, editors. Clinical Virology. New York: Churchill-Livingstone; 1997. p. 1133–86.

[5] Day JF, Stark LM. Frequency of Saint Louis encephalitis virus in humans from Florida, USA: 1990-1999. J Med Entomol. 2000;37:626–33. 10.1603/0022-2585-37.4.626 10916306

[6] Day JF, Curtis GA. Blood feeding and oviposition by*Culex nigripalpus* (Diptera: Culicidae) blood feeing and oviposition before, during and after a widespread St. Louis encephalitis epidemic in Florida. J Med Entomol. 1999;36:176–81. 10.1093/jmedent/36.2.176 10083754

[7] Day JF. Predicting St. Louis encephalitis virus epidemics: lessons from recent, and not so recent, outbreaks. Annu Rev Entomol. 2001;46:111–38. 10.1146/annurev.ento.46.1.111 11112165

[8] Shaman J, Day JF, Stieglitz M. Drought-induced amplification of Saint Louis encephalitis virus, Florida. Emerg Infect Dis. 2002;8:575–80. 10.3201/eid0806.010417 12023912 PMC2738489

[9] Epstein PR, Defilippo C. West Nile virus and drought. Glob Change Hum Health. 2001;2:105–7. 10.1023/A:1015089901425

[10] Curtis GA. Habitat selection strategies of mosquitoes inhabiting citrus irrigation furrows. J Am Mosq Control Assoc. 1985;1:169–73. 2906662

[11] Day JF, Curtis GA. When it rains, they soar—and that makes *Culex nigripalpus* a dangerous mosquito. Am Entomol. 1994;40:162–7. 10.1093/ae/40.3.162

[12] Fang Y, Reisen WK. Previous infection with West Nile or St. Louis encephalitis viruses provides cross protection during reinfection in house finches. Am J Trop Med Hyg. 2006;75:480–5. 10.4269/ajtmh.2006.75.480 16968925

[13] Ottendorfer CL. Impact of the West Nile virus on the natural history of St. Louis encephalitis virus in Florida [dissertation]. Tampa (FL): University of South Florida; 2008 [cited 2018 Oct 2]. https://scholarcommons.usf.edu/cgi/viewcontent.cgi?referer=https://www.google.com/&httpsredir=1&article=1437&context=etd

[14] Day JF, Stark LM. Avian serology in a St. Louis encephalitis epicenter before, during, and after a widespread epidemic in south Florida, USA. J Med Entomol. 1999;36:614–24. 10.1093/jmedent/36.5.614 10534957

[15] Komar N, Langevin S, Hinten S, Nemeth N, Edwards E, Hettler D, et al. Experimental infection of North American birds with the New York 1999 strain of West Nile virus. Emerg Infect Dis. 2003;9:311–22. 10.3201/eid0903.020628 12643825 PMC2958552

[16] Reisen WK, Chiles RE, Martinez VM, Fang Y, Green EN. Experimental infection of California birds with western equine encephalomyelitis and St. Louis encephalitis viruses. J Med Entomol. 2003;40:968–82. 10.1603/0022-2585-40.6.968 14765678

[17] Reisen WK, Lothrop HD, Wheeler SS, Kennsington M, Gutierrez A, Fang Y, et al. Persistent West Nile virus transmission and the apparent displacement St. Louis encephalitis virus in southeastern California, 2003-2006. J Med Entomol. 2008;45:494–508. 18533445 10.1603/0022-2585(2008)45[494:pwnvta]2.0.co;2 PMC2435167

[18] Centers for Disease Control and Prevention. Saint Louis encephalitis; 2015 [cited 2018 Jul 27]. https://www.cdc.gov/sle/technical/epi.html

[19] Centers for Disease Control and Prevention. Saint Louis encephalitis virus: epidemiology and geographic distribution; 2017 [cited 2018 Jul 27]. https://www.cdc.gov/sle/technical/epi.html

[20] Charosky L, Baldassari EL, Koren F, Mettler NE, Loizaga C. Síndrome encefálico a posible etiología por virus San Luis. Rev Asoc Med Argent. 1968;82:267–9.

[21] Mettler NE, Casals J. Isolation of St. Louis encephalitis virus from man in Argentina. Acta Virol. 1971;15:148–54. 4396413

[22] Spinsanti LI, Díaz LA, Glatstein N, Arselán S, Morales MA, Farías AA, et al. Human outbreak of St. Louis encephalitis detected in Argentina, 2005. J Clin Virol. 2008;42:27–33. 10.1016/j.jcv.2007.11.022 18249032

[23] Diaz LA, Ré V, Almirón WR, Farías A, Vázquez A, Sanchez-Seco MP, et al. Genotype III Saint Louis encephalitis virus outbreak, Argentina, 2005. Emerg Infect Dis. 2006;12:1752–4. 10.3201/eid1211.060486 17283629 PMC3372344

[24] Díaz LA, Albrieu Llinás G, Vázquez A, Tenorio A, Contigiani MS. Silent circulation of St. Louis encephalitis virus prior to an encephalitis outbreak in Cordoba, Argentina (2005). PLoS Negl Trop Dis. 2012;6:e1489. 10.1371/journal.pntd.0001489 22303490 PMC3269431

[25] Diaz LA, Nemeth NM, Bowen RA, Almiron WR, Contigiani MS. Comparison of argentinean saint louis encephalitis virus non-epidemic and epidemic strain infections in an avian model. PLoS Negl Trop Dis. 2011;5:e1177. 10.1371/journal.pntd.0001177 21629729 PMC3101189

[26] Rivarola ME, Tauro LB, Llinás GA, Contigiani MS. Virulence variation among epidemic and non-epidemic strains of Saint Louis encephalitis virus circulating in Argentina. Mem Inst Oswaldo Cruz. 2014;109:197–201. 10.1590/0074-0276130475 24810175 PMC4015247

[27] Diaz LA, Quaglia AI, Konigheim BS, Boris AS, Aguilar JJ, Komar N, et al. Activity patterns of St. Louis encephalitis and West Nile viruses in free ranging birds during a human encephalitis outbreak in Argentina. PLoS One. 2016;11:e0161871. 10.1371/journal.pone.0161871 27564679 PMC5001705

[28] Díaz A, Flores FS, Quaglia AI, Contigiani MS. Evaluation of Argentinean bird species as amplifying hosts for St. Louis encephalitis virus (Flavivirus, Flaviviridae). Am J Trop Med Hyg. 2018;99:216–21. 10.4269/ajtmh.17-0856 29761767 PMC6085794

[29] Calamari NC, Dardanelli S, Canavelli SB. Variaciones en la abundancia poblacional de palomas medianas a lo largo del tiempo. INTA EEA Paraná. Serie Extensión. 2011;64:23–8.

[30] Valinotto LE, Barrero PR, Viegas M, Alvarez López MC, Mistchenko AS. Molecular evidence of St. Louis encephalitis virus infection in patients in Buenos Aires, Argentina. J Clin Virol. 2012;54:349–51. 10.1016/j.jcv.2012.04.005 22608840

[31] Santos CL, Sallum MA, Franco HM, Oshiro FM, Rocco IM. Genetic characterization of St. Louis encephalitis virus isolated from human in São Paulo, Brazil. Mem Inst Oswaldo Cruz. 2006;101:57–63. 10.1590/S0074-02762006000100011 16699711

[32] Mondini A, Cardeal IL, Lázaro E, Nunes SH, Moreira CC, Rahal P, et al. Saint Louis encephalitis virus, Brazil. Emerg Infect Dis. 2007;13:176–8. 10.3201/eid1301.060905 17370543 PMC2725838

[33] Reeves WC, Hammon WM, Longshore WA Jr, McClure HE, Geib AF. Epidemiology of the arthropod-borne viral encephalitides in Kern County, California, 1943-1952. Publ Public Health Univ Calif. 1962;4:1–257. 14491029

[34] Murray RA, Habel LA, Mackey KJ, Wallace HG, Peck BA, Mora SJ, et al. Epidemiological aspects of the 1984 St Louis encephalitis epidemic in southern California. Proc Calif Mosq Vector Control Assoc. 1985;53:5–9.

[35] Reisen WK, Meyer RP, Milby MM, Presser SB, Emmons RW, Hardy JL, et al. Ecological observations on the 1989 outbreak of St. Louis encephalitis virus in the southern San Joaquin Valley of California. J Med Entomol. 1992;29:472–82. 10.1093/jmedent/29.3.472 1625296

[36] Tueller JE. An outbreak of illness due to St. Louis encephalitis virus in the southern San Joaquin Valley, California, 1989. Proc Calif Mosq Vector Control Assoc. 1990;58:12.

[37] McLean RG, Webb JP, Campos EG, Gruwell J, Francy DB, Womeldorf D, et al. Antibody prevalence of St. Louis encephalitis virus in avian hosts in Los Angeles, California, 1986. J Am Mosq Control Assoc. 1988;4:524–8. 2852209

[38] Hardy JL, Meyer RP, Reisen WK, Presser SB. A further evaluation of *Culex* mosquitoes in the greater Los Angeles area for their ability to vector St. Louis encephalitis virus. Proc Calif Mosq Vector Control Assoc. 1986;54:9–10.

[39] Reisen WK, Milby MM, Presser SB, Hardy JL. Ecology of mosquitoes and St. Louis encephalitis virus in the Los Angeles Basin of California, 1987–1990. J Med Entomol. 1992;29:582–98. 10.1093/jmedent/29.4.582 1495066

[40] Reisen WK, Lothrop HD, Chiles RE, Cusack R, Green EG, Fang Y, et al. Persistence and amplification of St. Louis encephalitis virus in the Coachella Valley of California, 2000–2001. J Med Entomol. 2002;39:793–805. 10.1603/0022-2585-39.5.793 12349864

[41] Kramer LD, Presser SB, Hardy JL, Jackson AO. Genotypic and phenotypic variation of selected Saint Louis encephalitis viral strains isolated in California. Am J Trop Med Hyg. 1997;57:222–9. 10.4269/ajtmh.1997.57.222 9288820

[42] Reisen W, Lothrop H, Chiles R, Madon M, Cossen C, Woods L, et al. West Nile virus in California. Emerg Infect Dis. 2004;10:1369–78. 10.3201/eid1008.040077 15496236 PMC3320391

[43] Feiszli T, Padgett K, Simpson J, Barker CM, Fang Y, Salas M, et al. Surveillance for mosquito-borne encephalitis virus activity in California, 2015. Proc Papers Mosq Vector Control Assoc Calif. 2016;84:124–9.

[44] Feiszli T, Padgett K, Simpson J, Barker CM, Fang Y, Salas M, et al. Surveillance for mosquito-borne encephalitis virus activity in California, 2016. Proc Papers Mosq Vector Control Assoc Calif. 2017;85:9–14.

[45] Saint Louis encephalitis virus (SLEV) in California counties 2017 YTD; updated Jan 2018 [cited 2018 Jul 27]. http://westnile.ca.gov/downloads.php?download_id=3839&filename=2017_slev_ county_map.pdf

[46] Chiu CY, Coffey LL, Murkey J, Symmes K, Sample HA, Wilson MR, et al. Diagnosis of fatal human case of St. Louis encephalitis virus infection by metagenomic sequencing, California, 2016. Emerg Infect Dis. 2017;23:1964–8. 10.3201/eid2310.161986 28930022 PMC5621550

[47] Arizona State Public Health Laboratory. Epidemiology and disease control. 2014 [cited 2018 Jul 27]. https://www.azdhs.gov/preparedness/epidemiology-disease-control/index.php.

[48] Venkat H, Krow-Lucal E, Hennessey M, Jones J, Adams L, Fischer M, et al. Concurrent outbreaks of St. Louis encephalitis virus and West Nile virus disease—Arizona, 2015. MMWR Morb Mortal Wkly Rep. 2015;64:1349–50. 26656306 10.15585/mmwr.mm6448a5

[49] White GS, Symmes K, Sun P, Fang Y, Garcia S, Steiner C, et al. Reemergence of St. Louis encephalitis virus, California, 2015. Emerg Infect Dis. 2016;22:2185–8. 10.3201/eid2212.160805 27869600 PMC5189155

